# Role of myocardial perfusion scintigraphy post invasive coronary angiography in patients with Myocardial Infarction

**DOI:** 10.4103/0972-3919.72687

**Published:** 2010

**Authors:** CNB Harisankar, Bhagwant Rai Mittal, KK Kamaleshwaran, Anish Bhattacharya, Baljinder Singh, Rajiv Mahajan

**Affiliations:** Department of Nuclear Medicine, Postgraduate Institute of Medical Education and Research, Chandigarh - 160 012, India; 1 Department of Cardiology, Postgraduate Institute of Medical Education and Research, Chandigarh - 160 012, India

**Keywords:** Acute myocardial infarction, MPS, revascularization, viability

## Abstract

**Background:**

The presence of severe hypokinesia or akinesia and near complete stenotic lesions on coronary angiography, in a patient with acute myocardial infarction raises a question of viability in the involved territory and its response to revascularization. The decision of revascularization can be effectively taken after myocardial perfusion scintigraphy (MPS).

**Aim:**

To evaluate the role of MPS in patients with acute or recent myocardial infarction after invasive coronary angiography.

**Materials and Methods:**

Thirty-five patients (27 Males, 8 Females; Mean age 54 years) with acute myocardial infarction, who underwent invasive angiography, were included prospectively. Invasive angiography was attempted during the episode of acute chest pain in 20 patients. Fifteen patients underwent angiography without MPS because of non-availability of MPS at the time of initial presentation in the referring hospital. Revascularization was deferred because of complete / near complete block of artery with hypokinesia / akinesia of the distal LV segments in 32 / 35 patients and 50 to 70% block in 3 / 35. These patients were subjected to MPS.

**Results:**

Twenty patients underwent stress MPS and 15 underwent nitrate-augmented rest re-distribution study (RR study). Imaging was performed using the hybrid SPECT / CT system. The average defect size of the perfusion defect was 34% (5 - 57% range). Sixteen patients (46%) had fixed perfusion defects. Reversible ischemia was present in 19 (54%). Ten patients had a < 10% reversible perfusion defect. Nine patients had reversible ischemia, > 10% of the LV myocardium, and underwent the invasive revascularization procedure.

**Conclusion:**

MPS is invaluable in patients who have total / near total occlusion of the coronary artery and distal segment hypokinesia or akinesia on invasive angiography. One in four patients, deemed to have non-viable myocardium, underwent an invasive revascularization after undergoing MPS.

## INTRODUCTION

Myocardial Infarction (MI) is a leading cause of death in developing countries. The ideal treatment modality for a patient with acute myocardial infarction has been accepted to be primary coronary intervention. Compared to thrombolytic therapy, treatment of patients with primary Percutaneous Coronary Intervention (PCI), at hospitals without on-site cardiac surgery, is associated with better clinical outcomes for six months after MI and a shorter hospital stay.[1] Thrombolysis, which is employed in managing the patients, requires that the patient present reasonably early to the hospital. Although early presentation of patients to the hospital is routine in the western world, it is not so common in developing countries. Many patients who would have benefited from early revascularization often present late to the hospital. Availability of expert care is another hurdle in the treatment of these patients.

Patients with ongoing chest pain often undergo coronary intervention in an attempt to revascularize the diseased coronary artery. The presence of severe obstruction of the coronary artery and akinesia of the myocardium distal to the site of stenosis, raises questions about the myocardial viability and hence, effectiveness of the revascularization procedure. It is well known that non-obstructing lipid rich plaques are often the culprits in acute coronary obstruction.[2] These often have thin fibrous caps, which, on erosion, expose the highly thrombogenic lipid material, causing acute thrombosis and hence myocardial infarction.[2] These can appear to be non-stenotic lesions when they undergo thrombolysis prior to invasive angiography. MPS is considered to be the gate keeper of invasive coronary angiography.[3] However, in a patient presenting with acute chest pain and other features of myocardial ischemia, revascularization is attempted as the initial modality. MPS may play a role in these patients following coronary catheterization. This study was conducted to evaluate the role of MPS in patients with acute or recent myocardial infarction after invasive coronary angiography.

## MATERIALS AND METHODS

We prospectively included 35 patients (27 Males, 8 Females) with a mean age of 54 years. All these patients fulfilled the following criteria

1. Documented acute myocardial infarction based on their clinical history, electrocardiographic changes, and biochemistry of cardiac enzymes.
2. They had undergone invasive coronary angiography.
3. The patients had not undergone coronary artery stenting. (The reason for not stenting the involved coronary artery during angiography was complete / near complete block of the artery with hypokinesia / akinesia of the corresponding LV segments in 32 / 35 patients and 50 to 70% block (hemodynamically equivocal plaque) in 3 / 35 patients).

The reasons for invasive angiography without prior MPS were ongoing chest pain at presentation (20 / 35 patients) and non-availability of MPS at the time of initial presentation in the referring hospital (15 / 35 patients). These patients were subjected to an electrocardiogram (ECG)-gated MPS as early as possible. The patients were subjected to physical exercise on a Treadmill (TMT) using the Bruce protocol whenever possible. One patient underwent an adenosine stress. When contraindications to stress were present (LVEF < 25%, LV aneurysm, LV clot, etc.), a nitrate augmented rest-redistribution study was performed.

### Stress protocol

A TMT stress test was preferred. The patients were given an initial warm up on the TMT at 1.0 mph and 0% inclination and were encouraged to walk for at least two minutes. The stress was changed to Bruce protocol after the initial period of warm-up.

Adenosine stress was given as per the standard protocol of 140 microgram/kilogram/minute for six minutes, with the tracer injection after three minutes of infusion. A nitrate augmented rest-redistribution study was performed after an intravenous injection of ^201^ Thallous chloride, three minutes after administration of 5 mg of sublingual nitrate.

### Acquisition

Images were acquired five minutes after the tracer injection in a hybrid SPECT / CT system (Infinia Hawkeye 4, GE healthcare, Milwaukee, USA) fitted with a low-energy, all-purpose collimator in a step-and-shoot mode, over 180 degrees. Attenuation correction was performed using an X ray source.

## RESULTS

The distribution of lesions in the individual coronary arteries were 28 in LAD, 12 in LCx,12 in RCA. On a patient-by-patient basis, 17 had single vessel disease, 10 had double vessel disease, and five had triple vessel disease, on angiography. Only three patients had stenosis < 70% in all the three coronaries. Fourteen patients had complete occlusion of the LAD with hypo- / akinesia of the territory supplied. Nineteen out of 35 patients underwent the TMT stress test. The average stress achieved was 5.8 METS with a range of 2.7 METS to 9.2 METS. The average heart rate achieved was 81% of the maximum permitted heart rate (Range 64% of MPHR to 100% of MPHR). None of the patients had severe or life-threatening side effects. One patient underwent Adenosine stress as per the standard protocol and did not have significant side effects. Fifteen out of 35 patients underwent the-nitrate augmented RR study.

**Figure 1:**
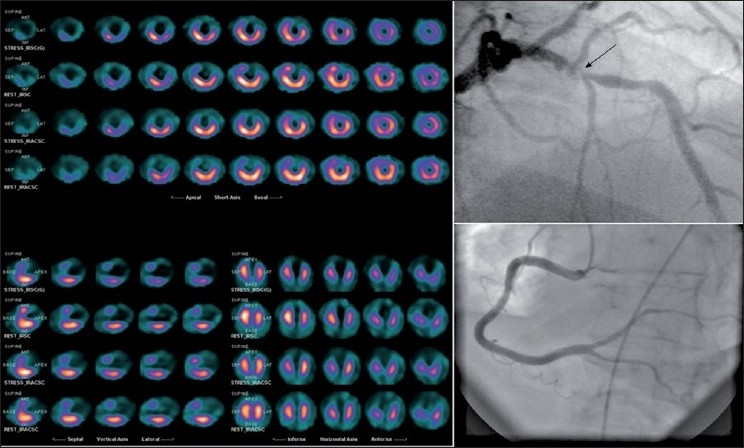
Myocardial perfusion scintigraphy showing an extensive fixed perfusion defect in the LAD territory, corresponding with the significant stenosis of the LAD on invasive angiography, (arrow). Revascularization was deferred in this patient because of the absence of a significant viable myocardium in the involved territory

**Figure 2:**
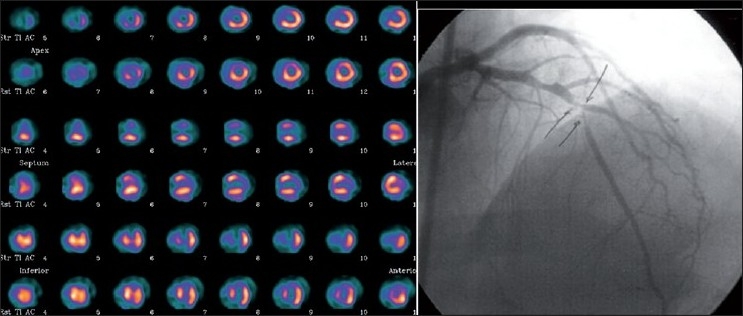
Myocardial perfusion scintigraphy in a patient with 90% LAD stenosis. Reversible ischemia involving > 10% of the LAD territory is noted. The patient was subsequently revascularized considering the significant viable, but ischemic myocardium in the LAD territory

The average defect size of the perfusion defect was 34% (5 - 57% range). Sixteen patients (46%) had fixed perfusion defects [Figure 1]. Reversible ischemia was present in 19 (54%) patients. Nine of the 35 patients (26%) had a greater than 10% reversible perfusion defect [Figure 2]. These patients underwent a second invasive angiography and revascularization procedure, due to the demonstration of a significant amount of ischemic myocardium. Of these nine, eight patients had 100% occlusion with severe hypokinesia of the LV in their previous angiography.

## DISCUSSION

The major question in a patient with total / near total occlusion of an epicardial coronary and akinetic myocardium, distal to the stenosis, is whether or not to proceed with coronary artery revascularization for improvement in symptoms and outcome. The identification of reversible myocardial dysfunction (i.e., hibernating or stunned myocardium) is an important finding in a patient with coronary artery disease.[4] Areas of hibernating or stunned myocardium contain preserved cell membrane integrity, despite abnormal myocyte contractility, and this contractibility can be restored after improvement in the blood flow. The presence of adequate amounts of viable myocardium can aid in the decision to proceed to a second coronary revascularization procedure, and hence salvage the myocardium at risk.

There is now a large body of evidence to support the use of various protocols using Myocardial Perfusion Imaging (MPI) to detect the viable myocardium and to predict improvements in the regional function in patients with ischemic LV dysfunction.[5,6] A 1997 meta-analysis by Bax and colleagues[5] found that all the radionuclide techniques, which included, MPI with rest / redistribution 201Tl, rest 99mTc sestamibi, ECG-gated SPECT sestamibi imaging, as well as stress / redistribution / reinjection 201Tl imaging, predicted an improvement in the regional and global LV functions, after revascularization. Using the quantitative analysis of the regional tracer uptake as a correlation of the magnitude of the viable myocardium, all myocardial perfusion techniques have similar negative and positive predictive values for predicting the improvement in the regional myocardial function. SPECT perfusion imaging with 201Tl and 99mTc sestamibi is slightly more sensitive than dobutamine echocardiography, but the latter has a higher specificity.[7]

The Investigating New Standards for Prophylaxis in Reduction of Exacerbations (INSPIRE) study[8] was one of the largest prospective trials yet completed, to use gated adenosine SPECT for the purpose of stratifying the risk, and thereby guiding therapeutic decision-making in clinically stable patients, early after AMI. These patients were those treated and stabilized after myocardial infarction. On the contrary, our patients underwent gated single photon emission computerized tomography (SPECT) for the purpose of identifying the correct management. All our patients had already undergone invasive angiography and could not be revascularized, either due to the status of the supplied myocardium (Akinesia or severe hypokinesia) or the supplying vessel (hemodynamically equivocal plaque). Furthermore, the cost of the study was grossly reduced by giving a physical stress test rather than an adenosine stress in our study.

The role of exercise testing, post myocardial infarction, for determining the prognosis of post myocardial infarction patients is well known. Patients who exercised early after an MI for 15 minutes or more were half as likely to suffer an event as those who did less. Furthermore, a long exercise time predicted an event-free survival.[9] Our study also elucidates the cost effectiveness of MPS in deciding the revascularization strategy in total / neat total occlusion of coronary arteries. If this strategy is followed to avoid stenting of totally occluded coronary vessels with akinesia / hypokinesia of the myocardium, 26% of such patients will be denied the opportunity of salvaging the viable myocardium. It is well known that the risk of cardiac death is several folds higher in patients harboring ischemic but viable myocardia, when compared to those with scarred myocardium.[10] On the contrary, if all the totally occluded vessels were stented, 75% of the patients would not have obtained any significant benefit. Furthermore, this would have greatly increased the financial expense of the procedure.

Gated SPECT not only identifies patients who are candidates for revascularization, but also offers prognostic information by way of evaluating the left ventricular ejection fraction (LVEF), lung uptake of the tracer.

## CONCLUSION

Myocardial perfusion scintigraphy is invaluable in patients who have total / near total occlusion of the coronary artery and distal segment hypokinesia or akinesia on invasive angiography. Twenty-six percent of the patients who would benefit from revascularization were identified with MPS. Furthermore, MPS prevented unnecessary stenting in 76% of these patients, by demonstrating the absence of viable myocardium in the involved territory. MPS was not only a simple and reliable study in this study group, but it also provided significant therapeutic and prognostic information on these high-risk patients.
